# Biobanks and Individual Health Related Findings: from an Obstacle to an Incentive

**DOI:** 10.1007/s11948-021-00330-9

**Published:** 2021-08-11

**Authors:** Jurate Lekstutiene, Søren Holm, Eugenijus Gefenas

**Affiliations:** 1 Institute of Health Sciences, Faculty of Medicine, Vilnius University, M.K. Čiurlionio 21, 03101 Vilnius, Lithuania; 2 Centre for Social Ethics and Policy, School of Law, University of Manchester, Manchester, UK; 3 Center of Medical Ethics, Institute of Health and Society, Faculty of Medicine, University of Oslo, Oslo, Norway

**Keywords:** Non-profit biobanking, Direct-to-consumer genetic testing, Return of individual health related findings, Ethical issues, Consent

## Abstract

Despite the benefits biobanks are expected to bring, there have recently been concerns raised that the public and private non-profit biobanks still prevailing in Europe often fail to reach their initial objectives due to a variety of reasons, including a shortage of funding and insufficient utilization of collections. The necessity to find new ways to manage biobanks has been clearly recognized and one way to do this is to follow the success of some commercial direct-to-consumer genetic testing (DTC GT) companies in the biobanking field. This paper is focused on a double role the return of individual health related findings (IHRF) detected through the biobanking activities can play in the management of biobanks. These findings can be seen as an untapped opportunity to offer health related information to biobank participants. At the same time, the IHRF policy can also serve as an additional tool that can improve biobanking governance. This paper aims to consider diverse IHRF approaches as well as to explore some key ethical concerns related to them. In particular, it reveals how different accounts of personal autonomy shape consent policies related to IHRF and emphasizes ethical controversies related to the commercial DTC GT initiatives as well as some non-profit biobanks.

## Introduction

During the last two decades biobanks have become an integral component of health research infrastructure. Although there is a great variety of biobanks, generally, research biobanks can be defined as an organized long-term repository of human biological material (HBM) associated with health-related data (HD) to be shared for future research (Kauffmann & Cambon-Thomsen, 2008; CIOMS 2016). In the following we will use the term ‘biobank’ to refer to research biobanks unless otherwise specified.

Biobanks can significantly contribute to the development of personalized medicine. They make high throughput scientific analyses possible by providing a large number of linked HBM and HD, enabling researchers to design and conduct studies that would otherwise be impossible. Combining different types of HD from large populations makes it possible to explore the complex relationships between genes and environment in the development of a disease. Using large numbers of biobanked HBM and HD, researchers can also identify disease genes, which are hoped to lead to early, more accurate diagnoses as well as individualized therapeutic and preventive options (Liu & Pollard, 2015; Zatloukal et al., 2018).

Despite the benefits biobanks are expected to bring, there have recently been concerns raised that the public and private non-profit biobanks (hereafter referred to as ‘non-profit biobanks’) still prevailing in Europe often fail to reach their initial objectives. This is due to a variety of reasons, including shortage of funding, low statistical power of collections and low sample numbers, and low utilization of collections (Chalmers et al., 2016; Kinkorová, 2016; Paradiso et al., 2018; Wai, 2012). These difficulties show that the initial optimism and expectations regarding their impact on progress in biomedicine were to some extent exaggerated. It might even be claimed that the initial hype that followed early biobanking developments was not justified and a metaphor of the “bubble burst” should be used to reflect the unfulfilled promises. The necessity to find new ways to manage biobanks has also been clearly recognized (Chalmers et al., 2016). To tackle these problems, biobanks join transnational biobank information networks (e.g., the European biobanking research infrastructure (BBMRI), the EuroBioBank). However, such networking might not reach its full potential due to very incongruent rules governing how HBM and HD are collected and used across different biobanks.

One important area of diverse provisions interfering with sharing of HBM and HD between different biobanks are regulations and policies regarding the return of individual health-related findings (IHRF) that can be of relevance to biobank participants. A requirement to comply with several different sets of regulations or policies can be an obstacle for biobank networking. Biobanks with diverse or even contradicting provisions related to the return of IHRF make it difficult for researchers to understand whether and what IHRF each biobank demands that researchers should convey to participants. In addition, such uncertainty may undermine the promises given to the biobank participants during the consent process.

A potential source of ideas on how to re-examine the role of IHRF and at the same time to facilitate biobanking activities could be drawn from a new biobanking model, which emerged in the last decade. This model has been introduced by commercial direct-to-consumer genetic testing (DTC GT) companies, which were predominantly established in the US. These for-profit companies have developed a business model, where, utilizing the latest technologies in genetic analysis, they offer paid DNA testing kits to the general population. These companies promise to reveal an individual’s ancestry, genealogy and even individual health risk factors. What is particularly important for our discussion is that through this service people buying a DNA test are also invited to provide their HBM and HD for future scientific research. Such a business model (which also includes biobanking) makes some commercial companies grow very fast. For instance, in the company 23andMe, most people (around 80 percent of all consumers) sign the consent form for biobanking (23andMe, 2020b). Therefore, just in 2018, nearly 5 million people gave their consent for future scientific research “generating an estimated $475 million in revenue for the company” (Sandler, 2020). Even though the demand for DNA testing kits seems to be decreasing, the availability of valuable data sets and the sale of these data to pharmaceutical companies and other partners may ensure the financial sustainability of some of these companies in the long run (Brodwin, 2018; Hamzelou, 2020).

Following the success of some commercial DTC GT companies in the biobanking field, one may wonder whether the management of non-profit biobanks should be reconsidered to ensure their long-term sustainability. Viewed from a slightly different angle, biobanks could become an alternative to the commercial DTC GT companies after addressing some important regulatory and ethical concerns. For example, in their early period of development these companies were severely criticized for manipulating people’s beliefs because they failed to separate between health and entertainment, offered genetic tests independent of physicians, and lacked regulatory oversight. In addition, they were accused of misguiding customers in relation to medical conditions, such as being a carrier of BRCA1 and BRCA2 genes, which may require an urgent intervention (Begley, 2019; Skirton et al., 2012). As a reaction towards this criticism some commercial DTC GT companies made an attempt to move towards a so-called DTC 2.0 model. This model is based among other criteria on a clearer separation between testing for health information and “infotainment”. It also integrates partnership with licensed medical professionals and offers DTC GT services only after regulatory approval. For instance, in 2017 23andMe gained the FDA’s approval to sell the first DTC test for genetic health risk (Allyse et al., 2018). Similarly, in 2019 another DTC GT company AncestryDNA—the main rival of 23andMe—also following the DTC 2.0 model started offering health tests by employing a physician remotely and promising not to monetise client samples and data when sharing them with third research parties (Ancestry, 2020, 2021a).^1^

It seems therefore that consumer/participant friendlier management of non-profit biobanks could also potentially encourage individuals to donate their HBM and HD for future research purposes. In this context it is important to re-examine a double role the return of IHRF detected through the biobanking activities can play in the management of biobanks. On the one hand, these findings can be seen as a not yet exploited opportunity to offer health related information for biobank participants that would not have the drawbacks of the commercial DTC GT services. On the other hand, the IHRF policy can also serve as an additional tool that can improve biobanking governance. Therefore, this paper aims at exploring some key ethical concerns related to the alternative models of IHRF and biobanking. In particular, it is important to reveal how different interpretations of personal autonomy shape policies of IHRF as well as to emphasize ethical controversies related to the consent procedures within commercial DTC GT initiatives as well as in some non-profit biobanks.

In order to assess these issues, non-profit biobanks and commercial DTC GT companies are analysed as case studies. Firstly, the importance of IHRF for biobanking is explored. Secondly, commercial DTC GT companies and non-profit biobanks are analysed as two different biobanking models. Such a comparison helps to emphasize different functions the return of IHRF have in each model. It also makes it possible to reveal shortcomings of the consent procedure offered by the commercial DTC GT companies, which can easily lead to misconception about the real aims of using consumers’ HBM and HD. Thirdly, the scope of IHRF to be reported to biobank participants, which is based on three approaches derived from the genetic testing context, is analysed and ethical issues related to these approaches, such as different accounts of personal autonomy and the role participants’ preferences play in defining the scope of IHRF, discussed.

## Importance and Terminology of IHRF

There are several reasons why it is necessary to reconsider the policies on IHRF. Firstly, due to increased use of next generation sequencing technologies, more and more findings are generated in the course of collecting HBM and HD by biobanks and later in conducting research. Secondly, people are interested in getting their IHRF. Thirdly, if IHRF are offered, individuals may have a stronger motivation to collaborate with biobanks by donating HBM and sharing HD (Viberg et al., 2016). This may increase the value of existing collections and also indirectly address other more general biobanking problems, such as insufficient utilization of collections (the higher the value of the collections, the greater likelihood that researchers will be interested in them). Also, if samples are used (which is what people actually expect when they donate) and important research deliverables are demonstrated, this would also make it easier to get funding for further biobanking activities. Last but not least, successful IHRF feedback policies offered by non-profit biobanks can serve as an incentive for individuals to switch from donating samples to the commercial DTC GT companies towards non-profit biobanks offering ethically justifiable IHRF policies.

Several types of IHRF that are relevant to biobank participants can be distinguished in the context of whole genome or exome sequencing. The terms “secondary findings” and “incidental findings” are used most often to refer to the findings that can be useful for a person but go beyond the primary indication for genetic testing. Although these terms are often used interchangeably, the term “secondary findings” seems to be more relevant in the biobanking context. Although it refers to the results that are unrelated to the primary objective of a biobank, that is collection of HBM and HD for future unspecified research, these results can nonetheless be systematically (rather than incidentally) sought out and analyzed when special filters are applied to the raw genetic information to identify what is regarded as “actionable” genes or other biomarkers.

Still another type of IHRF are the individual research results i.e., research findings concerning an individual research participant that have a potential health or reproductive importance and are discovered in the course of conducting biobank research.

Interestingly, commercial DTC GT companies declare IHRF to be a “primary” goal of their service. First of all, these companies promise to reveal some information about the individual and add a request to donate HBM and HD for biobanking purposes as a secondary optional objective. The authors agree that different types of IHRF can emerge in different circumstances that are related to biobanks in different ways and these contextual features are important for ethical analysis e.g., the ways they are generated in the biobanking activities or whether they are a primary or secondary objective for the providers. However, this differentiation is not so relevant when considering the scope and content of what should be returned to the biobank participant. Therefore, for the purposes of this paper the more generic term of IHRF will be used to cover all these different types of the findings.

## Non-Profit vs DTC GT Model of Biobanking and IHRF

Most European biobanks are non-profits (Beier & Lenk, 2015). Many of them have been established as part of a larger institution, such as a hospital, university or other private/public research or health care centre. The idea behind this was that researchers wanted to get easy access to linked HBM and HD. Their primary and in many cases the only purpose – to facilitate future research projects – is declared in the biobanks ‘policies, including the consent documents. The biobank consent processes are usually facilitated by the health professionals of the institution where the biobank is established. They consult an individual about the participation in a biobank and provide information sufficient to address the individual ‘s potential concerns. Initially when biobanks were established, they saw altruism and benefits for future generations as the main incentive to donate HBM and give access to HD (Simm, 2014). The only direct benefits that were offered for the biobank participant were the results of tests (like a blood test) done during the initial visit (Simm, 2014; UK biobank, 2010). However, due to increasing availability of next generation sequencing technologies, the list of important findings identified in the context of research and biobanking has also expanded. Thus, the moral duty of researchers to report findings has been increasingly recognized. This has already encouraged biobanks to re-consider their policies by offering some IHRF to biobank participants. However, many biobanks belonging to the BBMRI network still do not have policies on the return of individual health-related findings. Even if they do, those policies differ. Moreover, a significant part (21%) of those running biobanks (biobank directors, heads, managers or similar) who are supposed to know the existing legislation, still lack an understanding “if there were national laws or regulations mentioning the possibility of sharing individual results with biobank participants “ (Brunfeldt et al., 2018). In the meantime, many people are still sending their HBM and providing their HD to the commercial DTC GT companies in the hope of getting something valuable in return. To sum up, most European biobanks acting as non-profit entities still rely on altruistic donations coming from patients and healthy individuals. These biobanks usually promote a ‘cost recovery’ sharing with different partners. Their primary goal is biobanking, while the return of IHRF is a secondary goal, if it appears in the list of objectives at all.

On the other hand, the activities of the commercial DTC GT companies are based on the desire of consumers to receive information on their health status, ancestry, or genealogy. Their relationship with biobanking is more complex. In fact, the declared primary objective of 23andMe is the IHRF, while biobanking appears to be a secondary objective which can be refused by the individual. Therefore, in this case we can talk about a “reversed” biobanking model (a phenomenon of “secondary” biobanking) where biobanking appears as a “secondary” product of the primary goal of returning IHRF to a person buying this service. For instance, a company like 23andMe, operating mainly in the US, recruits individuals for biobanking through selling genetic ancestry and genetic health reports directly to consumers. While offering health and other information individually, this company also invites individuals to allow their HBM and HD to be used in future research. Therefore, the primary motive of individuals to donate their HBM and HD is not to facilitate the activities of health or other researchers, but rather to know more about their own health and genetics. For the clients of DTC GT companies biobanking remains more as a secondary “additional” issue. Such a perception is also supported by the informational policy of the DTC GT companies: the biobanking related terminology is almost completely lacking in the policy documents. For example, the term “biobank” is not mentioned at all on the current MyHeritage DNA Research Project consent form (MyHeritage, 2019). However, even though the term is used in another – 23andMe – company’s policy (the company even prepared a short biobanking consent form), different aspects of future storage and research are still hidden in the complexity of at least three consent forms related to research and biobanking. This makes it difficult for the participants to get a coherent perception on what biobanking and research really entail as compared to, for example, the non-profit large-scale UK biobank’s policy (UK biobank, 2010). In addition, in contrast to non-profit biobanks, the consent to biobanking is given online, most often without the presence of a health professional and in a non-health related environment. Therefore, it is not easy to ensure that all consent requirements are satisfied, although it should be admitted that using the online platform and apps (Illumina, 2012; Scott, 2012) for the interaction between the DTC GT company and consumer can make the consent process more dynamic and interactive. To sum up, the biobanking and the commercial use of the genetic information and health data is an important part of the long-term business model of DTC GT companies. However, the information related to the commercial aspect seems to be downplayed in the interaction with the consumers despite the use of some innovative elements of communication. Therefore, this can easily result in consumers’ misconception about the real aims of using their HBM and HD.

## Different Approaches to IHRF in the Context of Genetic Testing

As mentioned in the previous section, a majority of biobanks still do not have feedback policies. Those biobanks that have feedback policies often have contradictory rules about what, when and if findings “must, should, may, or must not be returned” (Thorogood et al., 2019). Therefore, the availability of these findings has also already created a considerable discussion among scholars as well as in international guidelines: how to handle such IHRF, particularly what should be the scope of IHRF to be returned to the biobank participants (Council of Europe, 1998; OECD Guidelines, 2009; CIOMS, 2016; Council of Europe, 2016).

In determining what IHRF may be of benefit to an individual, at least three different approaches to define the scope of IHRF can be distinguished in the context of genetic testing:

1. The medically actionable genes (MAG) approach. This approach is based on revealing predisposition to certain monogenic high penetrance disorders, which could be prevented or diagnosed and treated by health care interventions available. One example of such an approach is the American College of Medical Genetics and Genomics (ACMG) Recommendations for Reporting of Secondary Findings in Clinical Exome and Genome Sequencing.^2^ Although the Recommendations are primarily intended for use in the clinical context, they can also be relevant for genomic research. Following the last position of the ACMG, whenever a lab does whole genome or whole exome sequencing on a patient (regardless of the indication for which clinical sequencing was ordered), it should search for the selected 59 “actionable” genes on the list included in the Recommendations. When any clinically significant finding is detected, it should be reported to the ordering physician (Green et al., 2013; Kalia et al., 2017), unless a patient explicitly exercises the right not to know. This means that if a patient opted out of the 59-gene panel, the patient’s sequence at the relevant loci would not be analysed, meaning that there would be no interpretive findings for the lab to return to the clinician and patient. The 59 genes list, which is supposed to be regularly updated, includes only genes related to rare monogenic high penetrance pathogenic mutations causing 27 serious medical conditions in which the pathogenic genetic mutations might be asymptomatic for a long time. It is important to note that interventions are available to treat or prevent these conditions and this is what makes these conditions “actionable” according to the ACMG. It should also be noted that the initial ACMG position was rather paternalistically oriented as it did not provide the opportunity to refuse screening of the genes included in the predefined list of secondary actionable conditions (Green et al., 2013). Following on from this criticism, the ACMG changed its initial position by introducing the right to withdraw. (ACMG, 2014).
  A slightly different approach complementing the ACMG recommendations has been developed by Berg and colleagues (2016). These authors suggest not to rely on a specific (minimum) list of „actionable “genes, but rather provide an instrument to assist in determining the clinical actionability of gene–disease pairs. Following the suggested framework, Berg and his colleagues define five critical elements that need to be assessed to determine clinical actionability of a concrete genetic condition. These elements are: severity and likelihood of the disease outcome, efficacy and burden of intervention, and knowledge base, each having a score from 0 to 15. The higher the total score composed of these five components, the greater the actionability of the gene–disease pair. Similarly to the ACMG model, Berg and colleagues suggest to include only those genes that are associated with clearly defined monogenic disorders. However, their framework broadens the scope of returnable findings by including additional genes with scores equivalent to those on the current recommended ACMG list. This model seems to be more flexible as compared to the ACMG model as it „can be easily adaptable to different contexts by differential weighting of selected components" (Berg et al., 2016). However, it may also pose a serious problem in interpreting those five elements, as physicians and thereby biobanks may diverge on how they allocate scores to the categories. Therefore this model is currently better suited to facilitate „more efficient discussions and greater consistency than earlier attempts to arrive at consensus without a structured framework." (Berg et al., 2016). However, this model as well as the ACMG list of genes can still be considered as rather medically oriented ones because they do not integrate patients’ perspective.
2. The patient actionable genes (PAG) approach. Ploug and Holm, (2017) have developed another approach to determine the scope of IHRF. This is in contrast to the approach of ACMG and Berg and colleagues’ approach, which only targets “medically actionable genes. “ The MAG excludes conditions such as Huntington disease where no treatment options are currently available. These authors emphasize the importance of patients’ preferences and propose a shift from medically actionable genes to patient actionable genes (PAGs) (Ploug & Holm, 2017). Therefore, the latter approach is more congruent with the principle of personal autonomy as compared with the ACMG model. It includes untreatable conditions relevant for reproductive choices and therefore does not only focus on clinical actionability. As individuals have a great interest in information about genetic risk factors, Ploug and Holm suggest to introduce a model „based on a combination of population preferences and professional standards“. In other words, they argue that the scope of feedback should cover not only medically actionable genes, but also those where we cannot offer preventive measures and treatment but which person may have a strong interest in knowing never the less, namely „any variant with the following characteristics: (1) The variant is associated with a moderate or high risk (> 50%) of causing severe disease, (2) There is a high level of scientific evidence for the association, and (3) The manifestation of the disease is in the near to medium future.” This would, for instance, include variants that have significant impact for an individual’s future life plans (Ploug & Holm, 2017).
3. The DTC GT approach which includes multifactorial diseases. Most DTC GT companies follow an approach, which differs considerably from the first two described above. The success of commercial DTC GT companies is based on triggering human curiosity to explore genetic roots/ancestry/genealogy as well as to get information about health, including multifactorial as well as monogenic disorders. For instance, some DTC GT companies promise to inform the individuals whether they have certain gene variants increasing the risk of developing Parkinson's disease, Alzheimer's disease, breast cancer or diabetes. Although they search for a very limited number of genetic variants (e.g., in the case of BRCA genes), they claim to be analysing „some of the most well-studied variants, associated with extremely high risk” (Begley, 2019). At the same time, these companies are very careful to distinguish their services (health package) from the diagnostic process of identifying certain diseases. For example, they claim that their services are intended to provide results purely for informational, educational and research purposes. One of the reasons behind their claim is that neither the analytical methods, nor the relevance of the findings have been clinically validated. The technology that is used is mostly still the technology used in the research rather than the clinical setting (23andMe, 2020a; MyHeritage, 2020). Taking into account the complexity of the given information, the question remains whether people sufficiently understand what they receive when they decide to pay for such information. The issue of therapeutic misconception becomes a serious problem here. The term “therapeutic misconception” was first introduced to refer to the situation where research participants attribute therapeutic intent to research procedures (Appelbaum et al., 1982). However, the phenomenon of therapeutic misconception is also relevant in the context of biobanking. This is particularly the case when people expect to receive individual therapeutic benefits from their participation in the biobanking ignoring that its main goal is to establish an infrastructure for future research. Besides the issue of therapeutic misconception, data sharing seems to also pose serious problems to individual rights in the DTC GT companies. One medical journal has described it “as a ‘wild west environment’ of data sharing, lacking the rigorous regulation of the health care context“ (EDPS preliminary opinion 2020).

## Discussion

The three different approaches on how to define the scope of IHRF show a considerable variation of views among the experts on what type of IHRF should be returned to people donating their HBM and HD to biobanks. For instance, biobanks choosing the MAG approach suggested by the ACMG, and Berg and colleagues would employ a model only revealing predisposition to certain monogenic high penetrance disorders, which could be prevented or diagnosed and treated by health care interventions currently available. On the other hand, those opting for the PAG approach developed by Ploug and Holm would most probably also look at participants’ preferences when dealing with monogenetic diseases. Finally, many DTC GT companies promise to report IHRF related to both monogenetic diseases and some other IHRF related to risk factors for multifactorial diseases. Taking into account the diversity of the identified approaches, it seems to be important to explore them in terms of individual’s motivation and expectations as well as the utility of IHRF.

The DTC GT approach offering testing on different types of disorders might be the most motivating to donate HBM and HD, which is indicated by the high numbers of people donating their samples to these companies. This is also consistent with the results from studies of non-profit biobanks. For example, in one such study “over 80% of participants wanted to receive the genetic results regarding lifestyle diseases”, which is even higher than the number of those wanting to receive adult-onset-clinically actionable findings (Yamamoto et al., 2017). In another study, “more than half of respondents (57%) preferred disclosure even when there is uncertainty about the results’ meaning” (Allen et al., 2014). People’s motivation is also strengthened by the fact that the DTC GT service is easily and quickly accessible and usually no medical involvement is needed (Bollinger et al., 2013). What is more, a rather sophisticated language and terms provided in the online information (like “health reports”, “probability to get the disease”) can create the impression that something medically relevant can be given to all or at least most consumers.

However, what everyone get as a return is the confirmation of what everyone already knows. For example, everybody is more or less aware of his or her own family history and of certain health problems and risk factors that they have. For instance, that most (if not all) of us have a very low/medium risk of having or getting any of the multifactorial diseases, but that it is important to keep a normal weight and fitness level to reduce our risk, and we therefore need to be physically active, stop smoking and eat healthy food even without being tested. At the same time, it should be noted that although the DTC GT companies promise a variety of findings, they escape from legal liability as they claim that their services are not medical services but rather HD analyses done for educational, informational and research reasons. Perhaps this argument can work for the multifactorial diseases that are often caused by a multitude of genetic and environmental factors acting together, therefore it is difficult to make predictions for this type of diseases also in actual clinical practice. However, it would be more difficult to accept this line of argument in the case of monogenic diseases like BRCA1 or BRCA2. For instance, 23andMe selects only 3 of more than 20 variants to analyze BRCA genes, which will create both false negative results and some true positive results. However, it is difficult to maintain that a positive result is not a ‘medical’ result, because of its high predictive value for cancer, and a negative result is also likely to be interpreted as a medically important results by the consumer.

Both commercial DTC GT and non-profit research biobanks may rely on analytical methods that are sufficient for research purposes, but not generally considered sufficient for clinical purposes. They may for instance not routinely validate findings from gene arrays or sequencing. It is thus less certain that a ‘finding’ is really a finding or just an artefact or analytical error than it would be if the same result had been provided by a clinical testing lab. Most costumers and participants are probably not aware of this difference in the reliability of results, and if non-profit research biobanks begin to use feedback of genetic information as an incentivizing mechanism, they will have to provide accurate information about the reliability of this information to participants.

So, although the DTC GT approach may be the best motivator from the three options, it also seems to be the approach that misleads the consumers the most. Therefore, one may question if biobanks should put individuals into situations of choice where the risk of misconception is significant, and where findings of questionable value can pose serious psychological, social, economic or even physical harm. In addition, limited health care recourses are wasted due to unreasonable expectations from the tests made. Similar criticism would most probably also be expressed by the proponents of the biobanks following the „no return“ approach. For instance, a well-known population UK biobank after reconsidering the return of findings policy, decided not to provide individual biobank research findings and explicitly explained the reasons of not doing so in the consent form: “this is because such feedback outside of the normal clinical setting is of questionable value, and might even be harmful (for example, causing undue alarm and having potentially adverse effects on insurance status), especially when given without prior counselling or support." (UK biobank, 2010).

As for MAG and PAG approaches, returning IHRF is unlikely to increase individuals‘ motivation to participate in the biobanks as in the case of DTC GT approach. This is because the percentage of individuals who will have such findings is relatively small, from 1 to 3.5 percent (Evans et al., 2013; Bochud et al., 2017). However, these two approaches put different emphases on personal autonomy. The PAG approach puts significantly more „weight “ on what findings are seen as useful by people themselves as compared to the MAG approach. The MAG and PAG approaches do not create unreasonable expectations and do not push people to make health care decisions on inaccurate or incomplete information as compared to the DTC GT approach. The MAG approach may even decrease a person ‘s motivation to participate in a biobank in case it limits the right not to know (as was suggested in the initial position of the ACMG), because there may be a part of the population who have a strong preference for not being informed about even very serious IHRF. This would also generate a conflict between the values of individual autonomy and beneficence.

An interesting example of what IHRF should be reported is the Estonian Biobank. This biobank integrates some elements from each of the approaches described above. Besides promising what the ACMG listed in the MAG approach and what Ploug and Holm suggested in the PAG approach (e.g., carrier screening), the Estonian biobank also offers multifactorial disease risk estimates, e.g., of type 2 diabetes and coronary artery disease and information on drug response (BBMRI-ERIC, 2017). This approach is the closest to what the commercial DCT GT companies, particularly 23andMe, promise, although there are some differences as well. As compared to 23andMe, the Estonian biobank promises much more information related to the monogenetic diseases, and provides a more precise interpretation of findings. This is because it has a collaboration with the national health system, including full access to the e-health system, national registries and hospital databases. So this policy seems to be motivating people to donate HBM and HD. However, the question remains whether other biobanks should follow the Estonian policy on the IHRF because it is questionable how much the effect of such a policy is based on a therapeutic misconception. On the other hand, the notion of therapeutic misconception might be less controversial in the Estonian biobank as compared to some DTC GT companies, as the return of the IHRF in the non-profit biobanking brings biobanking infrustructure closer to health care where health care specialists can offer the interpretive help regarding the IHRF.

The feedback of non-medical genetic information, such as ancestry information, may incentivise without relying on a therapeutic misconception. The promise of providing ancestry information is very prominent in many of the adverts from DTC GT companies and may be the main reason for some people to use these services. However, this raises the issue of whether non-profit research biobanks should offer this kind of information and with what caveats. The main reasons for providing ancestry information would simply be that many people would like to have it, and that it might have a strong incentivising effect. The main reasons against is that the evidence base for precise genetic ancestry determination is weak, and that there might be a slippery slope (Blell & Hunter, 2019). The non-profits might start with ancestry information, but could then feel pressure to broaden their feed back even more to involve areas where the weak evidence base is more problematic because it may lead participants to take action on the basis of the feed back they get, e.g., nutrigenomics or the prediction of athletic ability (Pavlidis et al., 2016; Webborn et al., 2015).

Finally, it is still an open question whether it is good to drive individuals‘ motivation to participate in biobank activities by emphasising purely individual benefits. It is one case, when biobanks offer these benefits as an act of reciprocity and as an additional benefit, but clearly explains why research is important and why it is important to support research biobanking activities. It is, arguably another thing entirely when the participation in biobanks is incentivised solely by individual benefits As noted by Steinsbekk and colleagues (2013), such individualistic and ‘what’s in it for me’ attitudes could weaken feelings of altruism and be detrimental to “human conduct relating to contributing to biomedical research “.

Of note, recent biobanking activities find themselves more connected to health care systems and search for ways to combine both personal gains and common good. A few initiatives like the Estonian biobank aims to systematically link non-profit biobank research with the national health care systems through e-health and provide benefits of genomic analysis via the existing health infrastructure (ERR News, 2018). Other initiatives, such as the 100,000 Genome Project (UK) and All of Us Research program (US) have also been moving toward the merging of genomic analysis into the health care (Genomics All of Us, 2021; England, 2019).

Whether a person is motivated to donate for personal gain or for the common good, the most important thing, however, is to ensure and promote public trust in biobanking. As the scientific literature suggests, public trust toward biobanks positively impacts the willingness to donate (Domaradzki & Pawlikowski, 2019).

## Concluding Remarks

So far, the policies related to IHRF do not play any significant role in the biobanking field. This is because they are usually absent, or, if they exist, then they are unclear, vary across different biobanks and are often seen as an obstacle to the biobanking network. However, the authors believe that IHRF could legitimately be used as an incentive to donate samples more actively and to redirect donation of samples from the DTC GT companies to non-profit biobanks. Non-profit biobanks, whose primary purpose is to make it clear that everything is done for future research purposes, should be in a better position to counterbalance the drawbacks of feedback than the DTC GT initiatives. They could offer an alternative way of delivering IHRF that would be more ethically justifiable, better protect individual rights and be more beneficial to individuals.

To make this work, there are some important challenges to be met. One fundamental task is to pave the way for a more coherent approach on how to define the scope of the IHRF. Currently the scientific literature presents a considerable variety of criteria to define reportable (useful) findings. However, there is a lack of guidance on how to weight different approaches related to the scope and utility of the IHRF, particularly because their ethical assessments are different. This is especially true when dealing with multifactorial diseases, although these findings are increasingly reported within commercial DTC GT initiatives as well as some non-profit biobanks like the Estonian one.

Whichever approach would be taken as the guiding one, it is important to acknowledge that there may be financial, organisational, social, legal as well as ethical challenges that need to be tackled to make the value of IHRF work not only on paper, but also in practice. These issues require further analysis. In this context the particular ethical and legal challenge that arises for already existing biobanks in relation to IHRF needs close attention. Participants in these biobanks have consented to participation under a particular IHRF feedback policy, e.g., ‘no feedback’ or ‘only feedback of X, Y and Z’ and if the IHRF policies are to be harmonised between biobanks that might technically invalidate the original consent. In biobanks that have implemented a dynamic consent model^3^ this may not be a problem, but for most biobanks a solution has to be found which allows for the conditions of the original consent to be modified. This should involve dialogue with the existing participants to gauge their view on any changes to feedback policy and might therefore entail that some biobanks cannot join the ‘harmonised’ group since their participants are unwilling to modify the original policy.

## Footnotes

1 However, it should be noted that after 15 month effort to sell, the company decided to discontinue this service (Ancestry 2021b).

2 In the first versions of ACMG reccomendations, the term “incidental findings” was used. In 2016 ACMG shifted from the term “incidental findings” to “secondary findings”.

3 The term “dynamic consent” refers to a consent model that enables participants to become engaged about the use of their personal data through an interactive personalize online platform. This platform primarily aims to achieve two objectives: (1) facilitate the consent platform (a participant can change consent choices in real time) and (2) facilitate ongoing communication between researchers (biobank) and participants. (Budin-Ljøsne et al., 2017; Kaye et al., 2015).
